# Eye Gaze Patterns during Reasoning Provide Insights Regarding Individual Differences in Underlying Cognitive Abilities

**DOI:** 10.3390/jintelligence11040075

**Published:** 2023-04-20

**Authors:** Paulo Guirro Laurence, Tatiana Abrão Jana, Silvia A. Bunge, Elizeu C. Macedo

**Affiliations:** 1 Social and Cognitive Neuroscience Laboratory and Developmental Disorders Program, Center for Health and Biological Sciences, Mackenzie Presbyterian University, São Paulo 01241-001, Brazil; elizeu.macedo@mackenzie.br; 2 Psychiatry Program, Psychiatry Institute, Medicine School of São Paulo University, São Paulo 05403-903, Brazil; tatianaabraojana@usp.br; 3 Department of Psychology, University of California at Berkeley, Berkeley, CA 94720, USA; sbunge@berkeley.edu; 4 Helen Wills Neuroscience Institute, University of California at Berkeley, Berkeley, CA 94720, USA

**Keywords:** working memory, planning, executive functions, fluid intelligence, eye tracking

## Abstract

Sequences of eye movements during performance of a reasoning task has provided insights into the strategies individuals use to solve that specific task; however, prior studies have not examined whether eye gaze metrics reflect cognitive abilities in a way that transcends a specific task. Thus, our study aimed to explore the relationship between eye movement sequences and other behavioral measures. Here, we present two studies that related different eye gaze metrics in a matrix reasoning task with performance on a different test of fluid reasoning and tests of planning, working memory, and cognitive flexibility. Additionally, we related gaze metrics to self-reported executive functioning in daily life, as measured by BRIEF-A. To perform this, we classified the participants’ eye gaze in each item of the matrices test using an algorithm and then used LASSO regression models with the cognitive abilities as the dependent variable to select eye-tracking metrics to predict it. Overall, specific and distinct eye gaze metrics predicted 57% variance in the fluid reasoning scores; 17% variance in the planning scores; and 18% variance in the working memory scores. Taken together, these results support the hypothesis that the selected eye-tracking metrics reflect cognitive abilities that transcend specific tasks.

## 1. Introduction

Fluid reasoning (Gf; Carroll 1993) refers to a set of abilities that helps us solve new problems (Schneider and McGrew 2012). Gf supports relational and inferential reasoning, classification of new situations and phenomena, formulation of hypothesis, generalization, application of old schemas in new events and problems, and establishing similarities and differences between concepts (McGrew 2009). Gf refers a cognitive aptitude that relies heavily on working memory (WM), or the ability to keep relevant information in mind (e.g., Chuderski 2013; Conway et al. 2002, 2003; Dehn 2017; Engle et al. 1999; Kaufman 2014), as well as executive functions (EFs), or the set of control processes that support goal-directed behavior (Lehto 2004) and planning, or the ability to consider how to approach a complex problem before getting started.

**Figure 1:**
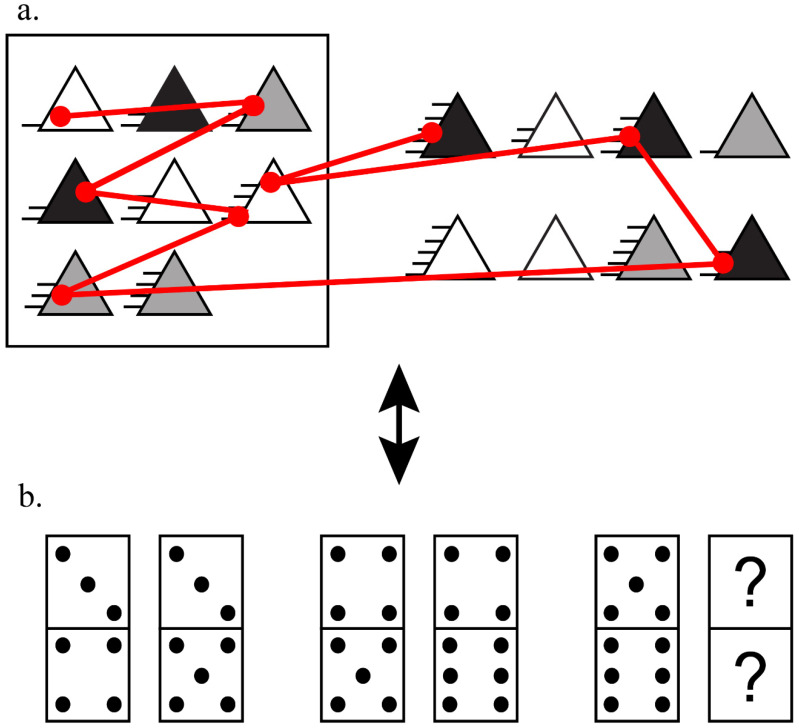
(**a**) Sample problem based on the WMT-2 task overlaid with a schematic representing an eye gaze pattern in red. Circles represent fixation locations, and lines represent eye movements, or saccades, between fixations; (**b**) sample problem on the D.70 test with the missing domino piece described as “?”.

Tests designed to measure Gf typically present a pattern of simple shapes, and the test taker must understand the rule that is guiding this pattern and then construct the answer or choose the correct answer in the answer choices bank (Alves 2007; Flanagan and Harrison 2012; Schlottfeldt and Malloy-Diniz 2018). One of the most common types of tests used to measure Gf are matrix reasoning tests, such as those found in Cattell’s Culture Fair task (Cattell 1973), Raven’s Progressive Matrices (Raven et al. 1998), the Wechsler Adult Intelligence Scale matrices (Wechsler 2004), and the Wiener Matrizen-Test-2 (WMT-2; Schlottfeldt and Malloy-Diniz 2018). These tests commonly present a 3 × 3 matrix with nine cells and a set of answer choices (see Figure 1 for a sample WMT-2 problem). All these tests are non-verbal measures that require inferring abstract relations among simple shapes and deducing the missing item in the array based on these relations. These types of tests assess deductive and inductive reasoning (Drozdick et al. 2012). Central to these tests is the fact that they require that participants identify and integrate relations (across the problem array), a cognitive process known as relational thinking (Alexander 2016) that was recently conceptualized as an EF (Starr et al. 2022).

When completing matrices problems, a test taker uses visual cognitive strategies. Vigneau et al. (2006) described two strategies in matrix reasoning tasks based on the work of Bethell-Fox et al. (1984), and Snow (1978, 1980) in analogical tasks with several response alternatives. The strategies can be defined as *constructive matching*, an effective but costly strategy whereby participants try to solve the problem by mentally constructing the missing piece before going to the answer choices to look for it; and *response elimination*, a less precise strategy whereby participants alternate many times between the matrix and the answer choices in an effort to eliminate wrong answer choices.

These two strategies, constructive matching and response elimination, can be identified through the use of eye tracking (Laurence and Macedo 2022). For example, the number of times a test taker’s eye gaze toggles between the matrix and the answer choices are proposed to be indicative of the strategy they are using: many toggles between them is theorize to reflect a response elimination strategy, while few toggles is thought to reflect constructive matching. The same arguments have been made for how long it takes for a participant to go to the answer choices for the first time after studying the matrix, or how much more time the participant spent on the matrix instead of the answer choices. Specifically, a long duration of focus on the matrix prior to transitioning to the answer choices, and proportionally more time spent analyzing the matrix than the answer choices, are thought to reflect use of the constructive matching strategy (see Laurence et al. 2018).

Further, algorithms have been developed to classify the scanpaths of different participants based on their similarities (Hayes et al. 2011; Kucharský et al. 2020). Through this approach, it is possible to use transition matrices on the areas of interest (AOIs), i.e., the matrix and the answer choices, and analyze this transition matrix to induce which strategy a participant had adopted (Kucharský et al. 2020).

These strategy measures have been shown to relate to performance on a matrix reasoning task. Previous studies established that these measures can predict performance on matrix reasoning tasks, demonstrating that participants with higher accuracy on the task tend to exhibit eye movement patterns similar to the constructive matching strategies (Laurence et al. 2018; Laurence and Macedo 2022; Vigneau et al. 2006). However, the eye gaze metrics in these studies were related to performance on the same task; we know of no studies analyzing the relation of the eye-tracking measures with a Gf score that was not measured in the same test where the strategy was measured.

Because WM is hypothesized to support the ability to reason about the relations among stimuli, several studies have investigated the relationship between self-reported strategy use and visuospatial WM. The relationship between WM and fluid reasoning is not new: several studies over the last 25 years have demonstrated that the capacity to solve new problems is directly associated with the capacity to keep relevant information in mind and manipulate it (e.g., Chuderski 2013; Conway et al. 2002, 2003; Dehn 2017; Engle et al. 1999; Kaufman 2014). However, research regarding the relationship between WM and cognitive abilities in the matrix reasoning task is much more recent (e.g., Gonthier and Roulin 2020; Jarosz et al. 2019). To assess strategies, most studies have either used questionnaires (e.g., Gonthier and Roulin 2020) or verbal protocols (e.g., Jarosz et al. 2019). These studies have demonstrated a relationship whereby higher visuospatial WM is related to the frequency of constructive matching strategy. In this case, participants appeared to use constructive matching for easy problems and resort to response elimination for hard ones. Further, the visuospatial WM predicted strategy use, with participants with higher WM scores maintaining the use of constructive matching strategy even for hard problems (Gonthier and Roulin 2020). It is noteworthy that, although this relationship between the type of strategy used and visuospatial WM has been reported in several studies, no study has investigated this relationship using eye tracking to quantify strategy use objectively, and on a trial-by-trial basis.

Since WM is related to the retention and manipulation of information, it is a limiting ability for most test takers (Gonthier and Roulin 2020). Besides visuospatial WM, which is important for keeping relevant visual features in mind, multiple other cognitive processes are thought to be required for performing the matrices test. For example, performing the test requires shifting between focusing on different elements of the matrix item, which can relate to the EF construct of cognitive flexibility (Birney and Beckmann 2022; Colzato et al. 2006). Further, performance likely benefits from a systematic approach to the problem (Cormier et al. 1990), i.e., planning. Strategy use on matrix reasoning can be related to these and other cognitive abilities; however, to the best of our knowledge, no study has tried to study the relations between them, especially with the use of eye tracking.

Fixation durations are also an eye behavior that is related to different cognitive abilities. First, individuals with high WM performance tend to exhibit longer fixations on the areas of interest of a distraction task (Luke et al. 2018; Meghanathan et al. 2015). Second, Hodgson et al. (2000) found that participants who performed well on a planning task called the Tower of London test showed shorter fixation times. This result indicates that good planners exhibit fast and efficient fixations. To the best of our knowledge, no studies have examined the relationship between eye gaze metrics and performance on a matrix reasoning task in relation to different cognitive abilities.

With these literature gaps in mind, our study aimed to explore the relationship between cognitive abilities and eye-tracking measures related to strategy use in matrix reasoning tasks. To perform this, we conducted two studies. In the first study, we measured reasoning on a computerized matrix reasoning task (Figure 1a) and a paper-and-pencil non-verbal inductive reasoning task (Figure 1b). In the matrix reasoning task, we recorded the eye movements of the participant and calculated metrics regarding cognitive strategy use. In the second study, we measured planning, visuospatial WM, cognitive flexibility, self-reported EF, and reasoning task performance with a matrix reasoning task.

We set out to test specific hypotheses regarding the relation of the eye gaze metrics on the WMT-2 reasoning task and individual differences in planning, WM, and cognitive flexibility. These hypotheses were tested specifically in Study 2. Our three key hypotheses and predictions, and our analytic approach, were pre-registered at AsPredicted (https://aspredicted.org/8sp6a.pdf, accessed on 14 April 2023).

First, we hypothesized that participants with better visuospatial WM would be able to retain matrix items and relations in mind more readily than others. Therefore, we predicted that visuospatial working memory would correlate with several gaze metrics. In particular, we predicted that the distinguishing characteristic would be the number of gaze transitions between the matrix and the answer choices. On this view, participants with better spatial working memory would be better able to keep in mind the features of a stimulus that would constitute the correct answer as they transition from the matrix to the answer choices. Additionally, they would be better to keep in mind an answer choice and check whether it fits, thereby making fewer transitions between the matrix and answer choices.

Second, we hypothesized that participants with superior planning ability would be more likely to adopt a constructive approach on the matrix reasoning eye-tracking task, spending more time observing the matrix problem than the answer choices. Thus, we predicted that planning ability would be associated with a higher proportion of time spent on the matrix vs. the answer choices.

Third, we hypothesized that participants with greater cognitive flexibility would perseverate less on the matrix reasoning task. Specifically, we predicted that the more flexible individuals, the ones with a low number of perseverative errors on the test of cognitive flexibility, would make fewer fixations to the incorrect answer choices on the matrix reasoning task, that is, they would not revisit irrelevant options multiple times.

We also sought to run exploratory analyses. We computed several strategy use metrics in the matrix test based on eye movements. Since we aimed to verify which of these metrics are related to different cognitive abilities, we used a feature selection method. We employed gold-standard methods of machine learning for small samples by using train/test split (see Vabalas et al. 2019) in order to select eye-movement predictors for reasoning task performance, planning, WM, cognitive flexibility, and self-reported EF in everyday thinking and behavior. All measures, with exception of the self-reported EF, are lab-based and objective measures, while the self-reported EF is a real-world, subjective measure. Therefore, it is noteworthy that in this exploratory analysis we tested the possibility that objective eye gaze metrics on an abstract reasoning task would be related to a subjective measure of real-world self-regulation. Furthermore, although we used methods for small samples, the samples presented in Study 1 and 2 are bigger than most of studies that investigated matrix reasoning strategies with eye tracking (see Laurence and Macedo 2022 for a list of sample sizes).

It should be emphasized that the feature selection method selected variables based on an algorithm. This selection is conditioned on the data. Although this type of feature selection can be used in databases with much more features than the size of the sample, the results should be observed with caution because this can lead to bias (Heinze et al. 2018); therefore, these exploratory analyzes should be analyzed with attention and criticism.

## 2. Study 1

### 2.1. Methods

#### 2.1.1. Participants

A total of 62 university students (40 women (66.12%), Median_age_ = 21, Range_age_ = 18–29) were recruited for this experiment, as part of a larger project. Two participants had an exceptionally low number of eye gaze fixations (two or fewer fixations per trial), which is under the threshold of 50% valid fixation data, and therefore were excluded from the study. The remaining 60 participants (39 women, 65%) ranged in age from 18 to 29 years old (M = 21.48, SD = 2.50). Data collection took place over three time periods: April to October of 2016; November of 2017 to April of 2018; and November to December of 2019.

#### 2.1.2. Instruments

**Eye-tracking matrix reasoning task (Figure 1a).** WMT-2 is a matrix reasoning test similar to Raven’s Progressive Matrices. It has a total of 21 problems, with three being examples that do not count to the final score (and were not analyzed) and 18 real problems. Items become more difficult as the participant progresses through the test. Each problem is composed of a 3 × 3 matrix, that is the problem, in the left and eight alternatives in a 2 × 4 matrix in the right of the screen. All items have two relations that participants must identify in order to select the correct answer. A sample problem similar to one found on the WMT-2 is presented in Figure 1. We used the computerized version of the test (Schlottfeldt and Malloy-Diniz 2018) and presented all stimuli in the sequence that the test guideline indicated. Between trials, a black fixation point was presented on a gray background for 2 s.

**Inductive reasoning task: D.70 (Figure 1b).** The D.70 test is a paper-and-pencil non-verbal inductive reasoning test that taxes visual, numeric, and basic numerical abilities (Chartier 2009). The test consists of 44 items that are a sequence of domino pieces with a missing piece. The participant’s objective is to draw the correct number of dots in a domino cell based on the pattern established by the sequence of numbers across the other pieces (see Figure 1). Participants were provided 25 min to try to solve all the items. We used the Brazilian version of D.70 (Alves 2007).

#### 2.1.3. Apparatus

To record the eye gaze data we used RED500 eye tracking from SensoMotoric Instruments, sampling at a temporal resolution of 500 Hz. We used iViewTM software (v. 3.7, SensoMotoric Instruments, Inc, SensoMotoric Instruments, Teltow, Germany) to calibrate the eye-tracking device and record the data, Experiment CenterTM (v. 3.7, SensoMotoric Instruments, Inc.) to present the stimuli, and BeGazeTM software (v. 3.7, SensoMotoric Instruments, Inc.) to extract the data. We used the default calibration procedure. The eye data algorithm used was the default of BeGazeTM, with a minimum fixation duration of 100 milliseconds.

#### 2.1.4. Procedure

The study was approved by the University Ethics committee (CAAE: 75035917.5.0000.0084). Participants were taken to the experiment room and seated at a desk. The experiment was explained to them, and if they agreed with their collaboration, they would provide their written consent. They first completed the paper-and-pencil D.70 test; next, they were placed in a chair ~70 cm away from a computer screen with a size of 19 inches width by 11 inches height. The eye-tracking calibration procedure was conducted. Participants were presented to the instruction screen of WMT-2, told how the test works, and had an opportunity to ask questions about it before beginning the experiment. Problems were presented in stimulus arrays spanning 23 × 13 cm. Participants were asked to provide their answers verbally, stating the number corresponding to one of the answer choices. The experimenter would write down their answer and proceed to the next trial. Upon completion of the study, participants received course credit.

#### 2.1.5. Eye-Tracking Measures

The average percentage of time that the eye tracker detected the eyes of the participant was 95.3% (SD = 4.13), with the participant that had the lower tracking ratio having their eyes detected 76.9% of the time in the task, while the participant with most tracking ratio presenting a tracking ratio of 99.8%. We excluded the first fixation in each trial, as well as all the fixations that were not in the matrix or answer choices. Additionally, we only used fixations with a duration over 100 ms since we were interested in cognitive fixations (Pieters and Wedel 2012). We calculated several eye-tracking metrics based on previous matrix reasoning studies (e.g., Laurence et al. 2018; Vigneau et al. 2006); we also calculated some common eye-tracking metrics and created new variables of interest (see Table 1 for the complete list of each variable), totaling 14 variables that were inserted in a multiple regression technique. Although 14 variables can be a lot for a standard regression model, we used a multiple regression technique involving data reduction, which is appropriate to avoid multiple comparisons.

In addition to computing these individual metrics, we used Kucharský et al.’s (2020) method to classify scanpaths, or the sequence of fixations over the course of a trial. This unsupervised method calculates a transition matrix based on the Areas of Interest (AOIs) for each scanpath on each trial. It uses standard *k*-means clustering, an algorithm based on lowering the within-cluster sum of squared Euclidean distances, to classify each scanpath into *k* clusters based on their Euclidian proximity. The *k* represents the number of centroids used to classify each scanpath. Based on the literature (e.g., Kucharský et al. 2020; Vigneau et al. 2006), we classified each scanpath using two centroids, following the idea of constructive matching and response elimination strategies. In other words, the scanpaths were classified into two possible unsupervised clusters. Following previous research (Hayes et al. 2011; Kucharský et al. 2020), we opted to delete repeated fixations in the same AOI. Further, we calculated the percentage of trials that a scanpath was classified into the second cluster for each individual. Since the percentage of trials that a scanpath was classified into the first cluster is a complementary measure of the percentage of scanpath classified into the second cluster, both measures can be employed for the model. The decision of which metric is used is purely arbitrary.

To compare the clusters and understand the differences between them, we calculated the Bayes Factor for each eye-tracking measure presented in Table 1. The BF_10_ is the Bayes Factor representing the strength of evidence for H1 over H0. By convention, values over 3 are considered moderate evidence in favor of the H1, values over 10 are considered strong evidence, and values over 100 are considered extremely strong evidence. On the other hand, values under 0.33, under 0.10, and under 0.01 are considered moderate, strong, and extremely evidence in favor of H0, respectively (Jeffreys 1961). In our tests, we considered H1 as a difference between the clusters for the gaze metrics, while H0 was considered as no difference between the clusters.

#### 2.1.6. Data Analysis

We used a Least Absolute Shrinkage and Selection Operator (LASSO) regression model to identify the subset of eye-tracking metrics that best predicted the D.70 test score. LASSO regression carries out the L1 regularization in the predictors by employing a penalty (*λ*) to the coefficients. This relationship of the coefficients and the shrinking parameter can be represented as:

(1) $$\|y-x\beta \|{}_{2}^{2}+\lambda \left\|\beta \right\|{}_{1}$$

With this feature, all coefficients are penalized. However, coefficients that have low predictive power are penalized until set to zero, while coefficients with high predictive power are penalized (down-weighted) but are not set to zero. This approach eliminates the variables with low predictive power and maintains the variables with higher predictive power. In our study, this method removed the eye movement metrics that are weakly associated with the cognitive measure. The shrinking parameter is user-selected, and because of it, it is necessary to perform cross-validation with several values of λ. To this end, we split our data into two parts: ~80% of the data (50 participants) were used to train the model and select the best penalty value based on the root mean square error (RMSE) with leave one out cross-validation; ~20% of the data (10 participants) were used to test the best model selected in the training dataset. To evaluate the model in the test set, we calculated performance estimates such as correlation coefficient (r); R^2^; mean absolute error (MAE); and root mean squared error (RMSE). Since the measures of the model are z-scores, the MAE and the RMSE present errors in standard deviations.

We also conducted a model using the same variables to predict the WMT-2 total score. This analysis used replicability and was compared with the model presented in this manuscript. The WMT-2 total score model is available in the supplementary file.

### 2.2. Results

#### 2.2.1. Descriptive

**Figure 2:**
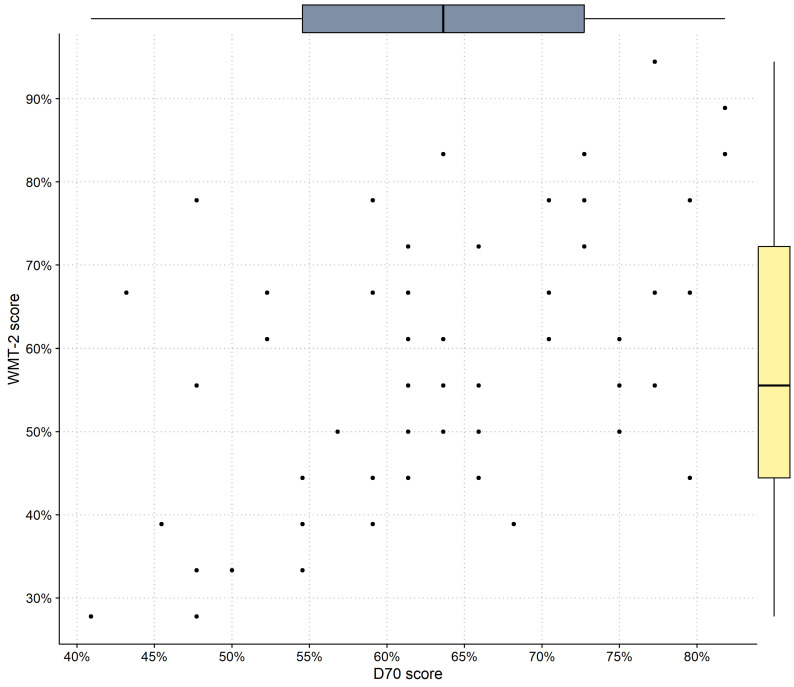
Scatter plot with box plots on the margin for the behavioral variables used in Study 1. The dots indicate the given test score of each participant, and the box plots present the data distribution for each variable.

Regarding the behavioral tests, the participants presented a mean (SD) percentage of correct answers of 63.41% (10.68) in the D.70 test (Skewness = −0.13, Kurtosis = −0.96). The percentages of correct items ranged from 40.91% to 81.82%. In the WMT-2 (Skewness = 0.05, Kurtosis = −0,94), the sample mean percentage of correct answer was 57.94 (16.78), ranging from 27.78% to 94.44%. Scores on the D.70 and WMT-2 tests were correlated, r = 0.54, *p* < 0.001. The descriptive of the behavioral variable used in this study can be found in Figure 2. Descriptive plots of the eye-tracking metrics are presented in the supplementary file.

The scanpaths for each trial for each participant were submitted to a classification analysis to identify different clusters of eye-movement strategies. In this analysis, two similar but separable strategies can be identified. The first cluster represents a strategy whereby participants explored the matrix row-by-row. The second cluster represents a strategy whereby participants explored the matrix row-by-row, but also in a column-by-column pattern (see Table 2). The transition matrix plots, the distribution of eye-movement metrics plots, and the description of the eye-tracking metrics for each cluster and for the eye-tracking metrics can be found in the Supplementary Material.

#### 2.2.2. LASSO Regression Model

Our LASSO regression model predicting D.70 performance from the eye-tracking metrics (shown in Table 1) performed well in the predicted direction, as judged by the performance estimates of the model (Table 3). A total of 12 variables were selected as contributing significantly to model prediction. Together, these variables predicted 57% of the variance in performance on the D.70 non-verbal inductive reasoning test.

## 3. Study 2

After noticing that the eye-tracking metrics derived from the WMT-2 were strongly related to performance on another visuospatial reasoning task, the D.70, we sought to test whether they would be related with other cognitive measures. We understand that WMT-2 and D.70 are different because they require a different set of abilities. For example, the WMT-2 provides answer choices, while the D.70 test requires you to create the missing answer. These differences may require different cognitive abilities, such as planning and visuospatial WM. Therefore, we were interested in understanding which eye gaze metrics were related to different cognitive measures. We conducted a second study with a new sample of participants to test for a relation between eye-tracking metrics on the matrix reasoning task and cognitive measures of planning, working memory, and cognitive flexibility, as well as self-reported EF. This study was pre-registered in the AsPredicted website (https://aspredicted.org/8sp6a.pdf, accessed on 14 April 2023). In this pre-registration, we wrote our intuitions regarding which cognitive measure would be related to the eye-tracking metrics. The rationale behind each hypothesis is: (1) Visuospatial WM positively correlates with the number of gaze transitions between the matrix and the answer choice since participants with higher WM can retain the matrix items in mind, which leads to fewer transitions. (2) Planning ability positively correlates with a higher proportion of time spent on the matrix vs. the answer choices since participants with higher planning abilities spend more time analyzing the matrix, trying to find patterns, and creating the missing piece. (3) The number of perseverative errors positively correlates with the number of fixations to the incorrect answer choices since participants that present higher perseverative errors are more susceptible to re-engaging in the same incorrect answer choice because of low cognitive flexibility.

### 3.1. Methods

#### 3.1.1. Participants

We recruited 73 participants (47 women, 64.38%, Median_age_ = 21, Range_age_ = 18–33) for this study. However, following our exclusion criteria mentioned in the pre-registration (i.e., <50% of eye-tracking data available, and/or 3 standard deviations from the mean score in the tower of London, Corsi block-tapping, Wisconsin Card-Sorting tests and the BRIEF-A global executive composite), 4 participants were excluded from the sample: three for poor eye-tracking data quality and one for a low score in the Tower of London test. Therefore, our final sample included 69 participants (45 women; 65.2%). Our sample had a mean age of 22.46 years (SD = 3.49), ranging from 18 to 33 years. All participants were university students. Most of them were students in law school (N = 31, 44.9%), followed by students in psychology (N = 23, 33.3%). Other students (N = 15, 21.7%) were majoring in engineering, physical therapy, pharmacy, architecture, economy, journalism, or neuroscience. Data collection occurred between February and November of 2017. Participants were recruited through social networks linked to the university and through the snowball sampling method.

#### 3.1.2. Instruments

**Figure 3:**
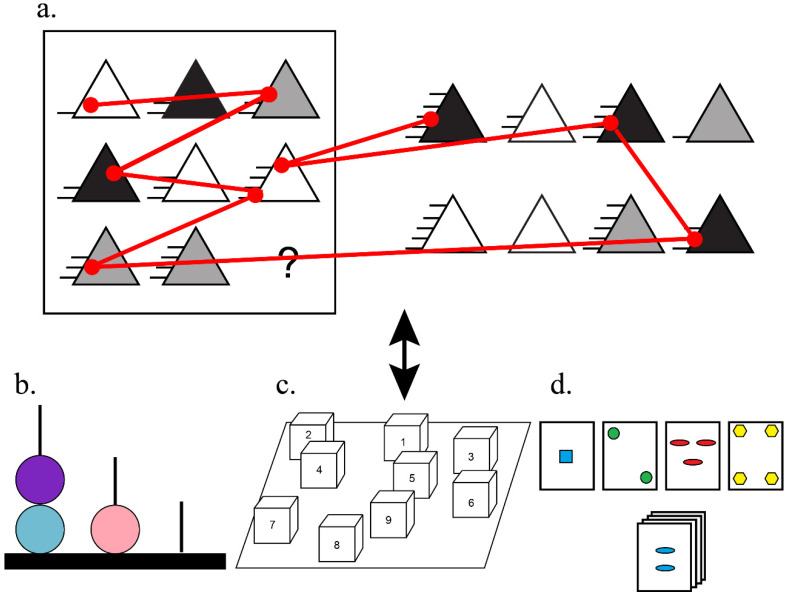
An example of (**a**) a WMT-2 item with an example of eye gaze (in red). The eye gaze metrics were correlated with the (**b**) Tower of London test, (**c**) Corsi block-tapping test, and (**d**) Wisconsin Card-Sorting test.

**Eye-tracking matrix reasoning task: WMT-2 (Figure 3a).** This is the same test described in Study 1.

**Visuospatial WM: Corsi block-tapping test (Figure 3c).** The Corsi block-tapping test is composed of a board with multiple blocks (Figure 3). It consists of two parts: in the first, the researcher touches the blocks in a sequence and the participant must repeat the same movements in that sequence; in the second part, the evaluator touches the blocks in a sequence and the participant must repeat the sequence inversely. The difficulty of the sequence increases with every two sequences made, with one more touch being added to the sequence. The test ends when the participant misses two sequences with the same number of **touches**. The total number of sequences is 14. The total test score varies between 0 and 28 (Corsi 1972; Santos et al. 2005), and higher scores indicate a better performance; we converted participants’ scores into percent accuracy. The Corsi block-tapping test was used successfully in the Brazilian context (Santos et al. 2005).

**Planning task: Tower of London (Figure 3b).** The Tower of London test assesses planning ability and logical reasoning. Participants must move balls in order to reach a target figure (Figure 3). The test has 12 target figures that participants should try to reach. At each level, the difficulty to reach the target figure increases. For each target figure, three attempts are allowed, and the answer is only considered correct if the solution is reached in the correct number of allowed moves. Thus, the score for each level ranges from 0 to 3, depending on how many times the participant has tried, and the total score ranges from 0 to 36 (Shallice 1982; Krikorian et al. 1994), with higher scores indicating a better performance. We transformed participants’ score into percentage accuracy. We also used the time score in the Tower of London as a cognitive measure.

**Cognitive flexibility: Wisconsin Card-Sorting test (WCST).** The Wisconsin Card-Sorting Test is an EF test in which the participant is presented with a sequence of 128 cards and must speak to which categorization criteria they are grouped. Criteria can be color, shape, or number of stimuli (see Figure 3). Criteria change after 10 hits in a row. This test can be evaluated by different types of measures, but we used perseverative errors, a measure of inhibitory control, and cognitive flexibility. In this case, higher scores in the perseverative errors indicate that they performed poorly. The test was adapted to the Brazilian context and can be used in this population (Heaton et al. 2004).

**Self-reported EF: Behavior Rating Inventory of Executive Function for Adults (BRIEF-A).** The BRIEF-A is a questionnaire that assesses self-regulation in daily life on adults aged 18 and older. It is 75 Likert-type items with three levels: “never”; “sometimes”; “often”. The items present statements such as “I have trouble with jobs or tasks that have more than one step” or “I make mistakes carelessly” and were created based on executive function concepts. The test has five questions for data validity that does not account for the final score. Therefore, the total BRIEF-A global executive composite (GEC) ranges from 0 to 210 and is provided by the sum of the behavioral regulation index (BRI) and metacognition index (MCI). The BRI is a composite score of inhibit, shift, and emotional control scale score, and ranges from 0 to 90, while the MCI is the sum of the initiate, working memory, plan/organize, organization of materials, and monitor scale scores, ranging from 0 to 120. A higher score on the BRIEF-A GEC and indexes indicates executive dysfunction (Roth et al. 2005; Roth et al. 2013). For the present study, we used the Brazilian version of BRIEF-A (Jana 2018).

#### 3.1.3. Apparatus

This is the same test used in Study 1. The data were recorded at a temporal resolution of 500 Hz.

#### 3.1.4. Procedure

The project was submitted to the Ethics and Research Committee and approved under CAAE number 63883016.0.0000.5487. Data collection was performed in a single session in the laboratory. Upon arriving at the laboratory, participants received explanations about the study and, if they wanted to continue with the participation, signed two copies of the Consent Form and Free Clarification. After that, the participants were taken to the room containing the eye-tracking equipment and were positioned approximately 70 cm away from a 19 inches width by 11 inches height monitor with the equipment for recording eye movements. The WMT-2 test was explained to the participant and then they answered the test. At the end of WMT-2, the BRIEF-A, Corsi block-tapping test, Tower of London Test, and WCST were applied. At the end of the procedure, the participant received course credit, a credit necessary for students to graduate, as a contribution to their participation.

#### 3.1.5. Eye-Tracking Measures

The average eye-tracking ratio was 95.7% (SD = 4.40). The participant with the lowest tracking ratio had 77.0% of the eyes detect in the task while the participant with the most tracking ratio presenting a tracking ratio of 99.7%. We used the same metrics used in Study 1. The full list of the metrics can be found in Table 1.

#### 3.1.6. Data Analysis

To test our hypothesis, we conducted several Steiger’s tests to compare the correlations of each predictor with the three cognitive test measures (Tower of London, Corsi block-tapping, and WCST perseverative errors). We opted to use Steiger’s test because we wanted to compare the correlations and see if one is more significant than the other regarding with each cognitive measure the eye-tracking metrics would correlate. Based on our hypothesis, we wanted to compare each of our hypothesized eye-tracking metrics with the cognitive tests. To calculate the Steiger’s test, it is necessary beforehand to calculate the correlation coefficients (r). Accordingly, we calculated the Pearson correlation coefficients between the three hypothesized eye-tracking metrics (i.e., ratio of time spent on the matrix vs. answer choices, number of matrix–answer transitions, and the average number of visits to a given incorrect answer choice) and the three cognitive tests. Steiger’s test statistically compares different correlations coefficients, without regard of the sign, in the same sample by calculating a z-value from the r, evaluating each difference with an asymptotic z-test, and then inferring the *p*-values. By convention, a significant difference between correlations coefficients w found when the test reveals a z-value greater than 1.96 in two-tailed tests, and therefore a *p*-value under 0.05 (Steiger 1980).

We also conducted seven LASSO regressions to select from all eye-tracking metrics, those, if any, that predicted the cognitive test measures and self-reported EF global score and indexes. We performed a data split on our sample. A total of ~80% of the data (57 participants) was used to train the model, and find the best value of the penalty, with a leave one out cross-validation, and ~20% of the data (12 participants) was used to validate the model. We evaluated our model in the test set and calculated the correlation coefficient, R^2^, the MAE, and the RMSE. As in Study 1, we also conducted a model with the same variables predicting the WMT-2 total score. The model is available in the supplementary file.

### 3.2. Results

#### 3.2.1. Descriptive

**Figure 4:**
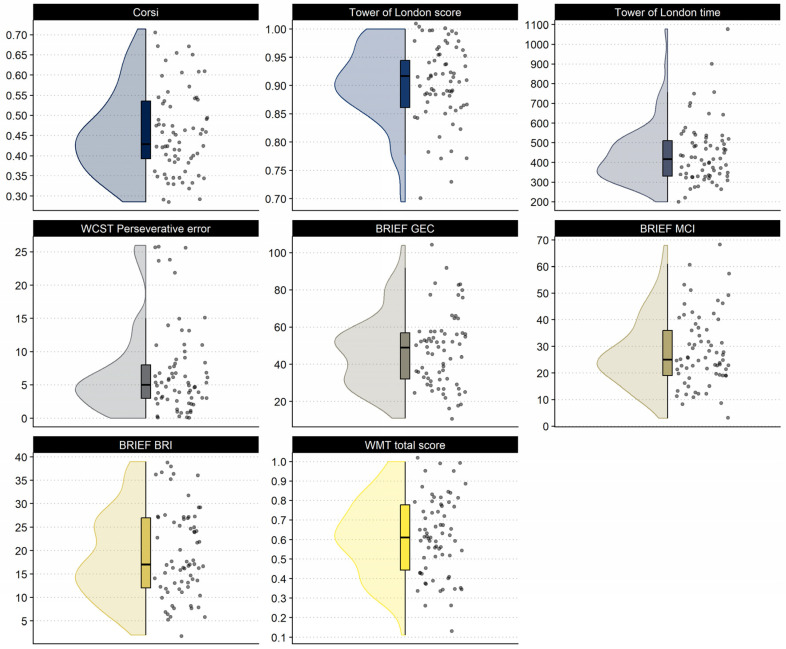
Raincloud plots for the behavioral variables used in Study 2. The dots indicate the given test score of each participant, and the box and violin plots present the data distribution. Note: All measures are in proportion correct of the total score, with exception of the WCST, which is the total raw number, the Tower of London Time, which is in seconds, and the BRIEF scores, which are in raw score. BRI: behavior regulation index; GEC: general executive composite; MCI: metacognitive index.

Regarding the behavioral data in Study 2, the participants in our sample had a mean (SD) percentage score of 45.61% (10.50), ranging from 28.57% to 71.42%, in the Corsi block-tapping test (Skewness = 0.52, Kurtosis = −0.49). In the Tower of London test (Skewness = −0.71, Kurtosis = 0.39), the sample’s percentage mean was 90.50% (6.89), with participants having a correct answer percentage between 69.44% and 100%. Their mean total time in the Tower of London test (Skewness = 1.50, Kurtosis = 3.21) was 444.03 s (155.28), ranging from 200 to 1078 s. Participants had, on average, 6.99 (6.63) perseverative errors in the WCST (Skewness = 1.58, Kurtosis = 1.87). The perseverative errors ranged from 0 to 26. Furthermore, our sample scored 47.26 (19.82) in the BRIEF-A general executive composite (Skewness = 0.54, Kurtosis = −0.14). The minimum score was 11 and the maximum score was 104. Regarding the BRIEF-A indexes, our sample had a mean of 28.19 (13.42) in the metacognitive index (Skewness = 0.71, Kurtosis = 0.17), and a mean of 19.07 (9.30) in the behavior regulation index (Skewness = 0.37, Kurtosis = −0.80). The indexes scores ranged from 3 to 68 and from 2 to 39, respectively. In the WMT-2 (Skewness = −0.07, Kurtosis = −0.63), the participants presented a percentage of items answered correctly mean of 61.44% (20.06). The percentages ranged between 11.11% and 100%. The descriptive of the cognitive tasks variables used in this study are presented in Figure 4. Descriptive plots of the eye-tracking metrics are presented in the supplementary file.

In the clustering analysis, both strategies were similar. The first strategy presented a pattern of following each row cell until going to the answers. The second strategy had a similar pattern; however, participants that used this strategy also had a pattern to follow the columns of the matrix. Participants who tended to adopt the second strategy had a higher probability to go to the answer choices from the end of each row, a pattern not found in the first strategy. The similarities and differences of both clusters are shown in Table 4. Plots of the transition matrix and of the distribution of different eye-movement metrics, and the descriptive of the eye-tracking metrics for each cluster can be found in the supplementary file.

#### 3.2.2. Comparing the Correlations

To investigate our hypothesis, we conducted a Steiger test between each of the predicting variables that we hypothesized and the three cognitive tests: Tower of London, Corsi tapping-block test, and WSCT perseverative error. To perform this, it is necessary to calculate the correlation coefficients beforehand (see in Table 5). It is noteworthy that no eye-tracking metric correlated with all three cognitive measures. However, the Tower of London score presented positive correlations with latency to the first fixation on an answer item (*p* = 0.002) and the ratio of time spent on the matrix vs. answer choices (*p* = 0.029) and was negatively correlated with average fixation duration for an answer choice (*p* = 0.036). The Corsi block-tapping test score was negatively correlated with the average time in each test item (*p* = 0.027), number of matrix–matrix transitions (*p* = 0.020), number of matrix–answer transitions (*p* = 0.002), number of answer–answer transitions (*p* < 0.001), number of visits to a given matrix cell (*p* = 0.011), number of visits to a given incorrect answer choice (*p* < 0.001), number of fixations on matrix cells (*p* = 0.011), and number of fixations on answer choices (*p* < 0.001). The WSCT perseverative error number correlated with the rate of matrix–answer transitions (*p* = 0.022).

**Figure 5:**
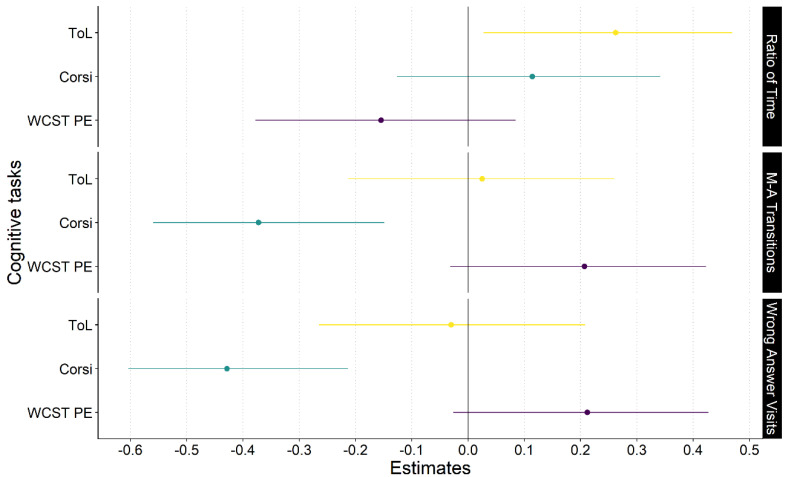
Forest plot presenting the correlation estimates and their 95% confidence interval for the correlation between the select eye gaze metrics and performance on cognitive tasks. The colors of the lines and dots are related to each cognitive test. Note: ratio of time: ratio of time spent on the matrix vs. answer; M-A Transitions: matrix–answer transitions; ToL: Tower of London test; Corsi: Corsi block-tapping test; WCST PE: Wisconsin Card-Sorting Test Perseverative Errors.

To analyze the relation of the eye-tracking metrics with each cognitive test and evaluate whether there was a statistical difference between them, we performed a Steiger test for each eye-tracking metric (see Figure 5). On one hand, the correlations of the ratio of time spent on the matrix vs. answer metric and the three cognitive tests presented a non-significant *p*-value, z = 1.74, *p* < 0.08. On the other hand, the correlations of matrix–answer Transitions metric with the three cognitive tests presented significant differences between them, z = 2.2, *p* < 0.03. Lastly, the correlations of the wrong answer visits metric with the three cognitive tests presented no significant difference, z = 0.32, *p* < 0.75.

#### 3.2.3. LASSO Regression Models

From the seven proposed LASSO regression models, four were able to find predictors for the dependent variable. The coefficients of each selected variable and the model measures are displayed in Table 6. The Visits in the wrong answer choices and the percent of trials classified as cluster 2 scanpath were able to predict 18% of the variation in the Corsi test. Latency of the first fixation in answer choices, the ratio of time spent on the matrix vs. answer, and mean fixation duration in the matrix predicted 16% of the variation in the Tower of London score. Although Tower of London time score had several predictors, its R^2^ was negative, indicating that the model was not reliable. The model with WCST perseverative errors as the dependent variable did not identify any predictors. Lastly, a combination of four eye-tracking metrics were selected in the training model for the BRIEF-A BRI. However, the test model presented a negative R^2^ (−5%), meaning that it was not a reliable model. No predictors were identified for the BRIEF-A MCI or GEC.

## 4. General Discussion

Our study aimed to explore the relationship between cognitive abilities and eye-tracking metrics related to strategy use in matrix reasoning tasks. Our preregistered analyses revealed relationships between several eye-tracking metrics with different cognitive abilities. We predicted that the ratio of time spent on the matrix vs. the answer choices would be more related to planning than the other cognitive measures (Hy1). Indeed, we found a *p*-value lower than 0.1 in this hypothesis, pointing to the possibility that planning is reflected in more time spent gazing on the matrix, but it was not significant. We also predicted that fewer gaze transitions between the matrix and the answer choices would be related to higher visuospatial WM scores (Hy2); this prediction was confirmed. Lastly, we predicted that fewer perseverative errors on WCST would be more related to less revisit in incorrect answer choices; however, this hypothesis was not confirmed. Based on Steiger’s test, no statistical significance was found pointing to difference between correlations of the cognitive measures. In summary, we were able to confirm our hypothesis that fewer gaze transitions between the matrix and the answer choices were related to higher visuospatial WM scores and found a low, yet not-significant, *p*-value indicating that planning can be related to more time spent gazing on the matrix. However, we also predicted that fewer perseverative errors on WCST were related to less revisits in incorrect answer choices, but this was proven not to be true.

We also conducted exploratory analyses to investigate the relationship of the eye gaze metrics with the cognitive tests. The test that was most similar to the eye-tracking task (another visuospatial reasoning task) was the one well-predicted by eye gaze metrics. The results in Study 1 showed a strong relationship between several eye gaze metrics and the D.70 score. These variables predicted 57% of the variation in the D.70 test. These results are consistent with previous literature (e.g., Hayes et al. 2011; Laurence et al. 2018; Vigneau et al. 2006), in which the eye gaze metrics predicted the participant score in the same reasoning task. With our results, it seems that the eye gaze metrics in a reasoning task can also predict the participant score in another reasoning task. Therefore, it is possible that the eye gaze metrics have a relationship with the reasoning ability of a participant.

Eye gaze metrics were also a moderate predictor of planning scores (16%). Higher scores on the Tower of London, a cognitive measure of planning, were related to higher Latency to the first fixation in answer choices, a higher ratio of time spent on the matrix vs. answer, and a smaller mean fixation duration in the matrix. We reasoned that participants that show better planning abilities are the ones that would first try to solve the problem on the matrix and then go to the answer choices, suggestive of a constructive matching strategy (Hy1). With this in mind, the eye gaze metrics selected were all related with the constructive matching: a high latency to the first fixation in answer choices points out that participants were scanning the matrix before going to the answer; a higher ratio of time demonstrates that participants spent more time fixating in the matrix than in the answer choices. Shorter fixations were also related to planning. Hodgson et al. (2000) demonstrated that participants who showed better performance in the Tower of London test were the ones with lower fixation times, suggesting that good planning is linked to fast and efficient fixations. However, the eye-tracking metrics were not good predictors for planning time, indicating that their time and efficiency are mediated by other variables.

Further, higher scores on the visuospatial WM task (Corsi block-tapping) were related to fewer visits to wrong answer choices and the percent of trials classified as cluster 2 scanpath (18%). While not predicted a priori, this result suggests that participants with higher WM are better able to remember elements of the problem, and therefore visit the wrong answer choices fewer times. We predicted higher WM scores to be related to fewer matrix–answer transitions (Hy2), based on previous work that demonstrated that individuals with higher WM used the constructive matching strategy more times (Gonthier and Roulin 2020). However, the percent of trials classified as cluster 2 scanpath was a negative predictor, indicating that participants that relied more on an eye gaze strategy that is very similar to constructive matching were able also more probable to present high visuospatial WM scores. Additionally, we predicted that visits in wrong answers would be related to perseverative errors in the WSCT (Hy3); however, this hypothesis was not borne out either. A possible explanation for this is that our sample had relatively small variation in the WCST perseverative error measure (Figure 4). Perhaps in a more diverse sample than university students, variation in perseverative errors would be higher and it would be possible to observe a relationship.

It is interesting to note that the rate of matrix–answer transitions was the best predictor for several studies predicting the score in the same matrix reasoning task that the eye gaze was recorded (e.g., Hayes et al. 2011; Laurence et al. 2018; Vigneau et al. 2006). We found similar results when predicting the score of another reasoning task, the D.70 test. However, this variable was not selected in any model predicting the score of the other cognitive tests related to visuospatial working memory, planning, and cognitive flexibility. The lack of relationship between the rate of matrix–answer transitions with other cognitive measures besides reasoning indicates that this metric is extremely related to reasoning, but not to other cognitive abilities. It is hard to understand precisely why this metric is related to reasoning ability, but it seems to be a reliable predictor of reasoning, even when predicting reasoning in a different task.

No predictor was found for the BRIEF-A general executive composite and metacognitive index. However, we were able to find predictors for the BRIEF-A behavior regulation index. In this case, ratio of time spent on matrix vs. answer choices, number of fixations in the matrix, mean fixation duration in answer choices, and average time in each test item were selected as predictors. However, in the test split, we found a negative R^2^ (−3%), indicating that these measures are not reliable to predict BRIEF-A BRI. These results indicate that self-reported daily EFs are not related to the eye gaze metrics in the reasoning task. Therefore, these real-world, subjective measures are not predicted by the lab-based, objective measures.

Regarding the clusterization of the eye gaze in the matrix reasoning task, we chose, based on previous literature (e.g., Kucharský et al. 2020; Vigneau et al. 2006), to use two centroids. This number of centroids was selected based on the idea of constructive matching and response elimination strategies. In both studies, we found a cluster that is very similar to the constructive matching pattern and another cluster that had a row-and-column-wise scan, with more transitions to the answer choices. This pattern was also verified by Kucharský et al. (2020), indicating that this pattern can be seen in different studies with the clusterization with two centroids. Indeed, we expected to find a second pattern that was more similar to the response elimination strategy but found an eye gaze pattern that is not a pure response elimination, but is also not a constructive matching pattern, although it has some similarities to it. Previous studies demonstrated that the pure response elimination strategy is very rare, with participants performing it in less than 3% of the trials (Jarosz et al. 2019). It was more common for test takers to execute a hybrid strategy. Therefore, our second cluster may reflect this: a strategy that has elements of the response elimination strategy (i.e., more transitions to the answer choices), but that also follows elements of the constructive matching strategy (i.e., the row-and-column-wise scan).

Differences found in the Bayesian post-hoc comparisons of the clusters indicated that, in Study 1, the constructive matching (cluster 1) scanpath had longer fixations than cluster 2, while the inverse was true in the Study 2. However, it is noteworthy that the biggest difference between the studies were in the second cluster. This indicates that the first cluster had a small variation while the second cluster presented a higher heterogeneity. These result patterns indicate that the second cluster can present distinct patterns since it is a more chaotic strategy, similar to the strategy described by Jarosz et al. (2019). It is noteworthy that differences in the clusters post-hoc analysis can be due to the clusterization itself. This means that since the clusters are generated by different features in eye gaze, these differences can also be related to the fixations and the other eye gaze metrics. Taking this into account, these results are very provisional.

The present study has broader implications for understanding the relationship between eye movements in matrix reasoning tasks and cognitive abilities. It demonstrates that the cognitive strategies in matrix reasoning tasks, measured by eye tracking, have a moderate relation with cognitive abilities of planning and WM. In this case, it is possible to think of how cognitive abilities intervention for WM or planning can affect the eye gaze on the matrix test. Additionally, studies teaching participants how to process the matrix in an efficient way may produce cognitive gains. New studies seeking to answer these questions are needed.

There are several limitations of this study to consider. First, we had two different samples with different cognitive measures instead of one with all the cognitive measures. Second, both samples consisted of university students, which limits the generalizability of the results. Future studies should focus on diverse samples. Furthermore, our sample size was relatively small, and our results should be interpreted with caution. Due to the sample size, both of our studies are underpowered (67% in Study 1 and 74% in Study 2), although both studies present bigger samples than most eye-tracking studies in this type of research (see Laurence and Macedo 2022). New studies should focus on larger samples.

## 5. Conclusions

To conclude, we aimed to explore the relationship between cognitive abilities and eye-tracking metrics related to strategy use in matrix reasoning tasks. The Gf test was the one better predicted by the eye-tracking metrics. After the Gf test, the WM and planning tasks were also the ones that the eye-tracking metrics predicted higher variance. This pattern of results supports the claim that the cognitive visual strategies used in the matrix reasoning task are influenced by cognitive abilities such as fluid reasoning, WM, and planning.

## Figures and Tables

**Table 1:** Eye gaze metrics used in this study.

	Eye-Tracking Metrics
1	Average time in each test item
2	Number of matrix–matrix transitions (number of times that a participant gazed from a matrix cell to another matrix cell)
3	Number of matrix–answer transitions (number of times that a participant gazed from the matrix to the answer choices or vice versa)
4	Number of answer–answer transitions (number of times that a participant gazed from an answer choice to another answer choice)
5	Latency to the first fixation on an answer choice (the time it took for a participant to perform the first fixation on the answer choices)
6	Ratio of time spent on the matrix vs. answer choices (time spent on the matrix divided by the time spent on the answer choices)
7	Average number of visits to a given matrix cell (the mean of the number of visits to each cell in all test items)
8	Average number of visits to a given incorrect answer choice (the mean of the number of visits to each answer choice, excluding the correct choice, in all test screens)
9	Total number of fixations on matrix cells
10	Average fixation duration for a matrix cell
11	Total number of fixations on answer choices
12	Average fixation duration for an answer choice
13	Percent of trials classified as cluster 2 scanpath (the percent of the items that the participant had their eye gaze classified as the cluster 2 scanpath)
14	Rate of matrix–answer transitions (the number of matrix–answer transitions divided by the average time in each test item; this conversion equalizes the number of matrix–answer transitions by how much time each participant spent gazing at each item. Higher rate indicates that participants gazed more times their eyes between the matrix and answer choices per second)

**Table 2:** Comparison of the model-based clusters of eye movements. The comparison was conducted based on the transition matrices or the Bayes Factor for each cluster.

Metric	Cluster 1	Cluster 2	Analysis
Gaze direction	Row-wise	Row-wise and Column-wise	Transition matrices (see supplementary file)
Probability of transition to answer choices from top or middle row	Low	Low to moderate	Transition matrices probabilities (see supplementary file)
Average time in each test item	-	-	BF10 = 0.07 (±<0.00) ^oo^
# Matrix–matrix transitions	-	-	BF10 = 0.21 (±<0.00) ^o^
# Matrix–answer transitions	-	-	BF10 = 0.08 (±<0.00) ^oo^
# Answer–answer transitions	More transitions	Fewer transitions	BF10 = 10.19 (±<0.00) **
Latency to the first fixation on an answer choice	-	-	BF10 = 1.09 (±<0.00)
Ratio of time spent on the matrix vs. answer choices	-	-	BF10 = 0.07 (±<0.00) ^oo^
# Visits to a given matrix cell	-	-	BF10 = 0.17 (±<0.00) ^o^
# Visits to a given incorrect answer choice	-	-	BF10 = 0.46 (±<0.00)
# Fixations on matrix cells	-	-	BF10 = 0.07 (±<0.00) ^oo^
Average fixation duration for a matrix cell	Longer fixations	Shorter fixations	BF10 > 1000 (±<0.00) ***
# Fixations on answer choices	-	-	BF10 = 0.38 (±<0.00)
Average fixation duration for an answer choice	Longer fixations	Shorter fixations	BF10 > 1000 (±<0.00) ***
Rate of matrix–answer transitions	More transitions per second	Less transitions per second	BF10 = 8.13 (±<0.00) *

Note: H1: difference between the clusters; H0: no difference between the clusters. * Moderate evidence for H1; ** strong evidence for H1; *** extreme evidence for H1; ^o^ moderate evidence for H0; ^oo^ strong evidence for H0.

**Table 3:** Coefficients and measures of the LASSO regression model predicting the D.70 total score.

Measures	Standardized Coefficients
Predictors ^1^	
Average time in each test item	−1.68
Matrix–answer transitions	1.40
Answer–answer transitions	1.80
Latency to first fixation in answer choices	−0.09
Ratio of time spent on matrix vs. answers	−0.25
Visits in wrong answer choices	−2.61
Total number of fixations on matrix cells	0.84
Average fixation duration for a matrix cell	−0.07
Total number of fixations on answer choices	−0.01
Average fixation duration for an answer choice	0.39
Percent of trials classified as cluster 2 scanpath	0.15
Rate of matrix–answer transitions	−1.10
Performance estimates	
Correlation coefficient	0.77
MAE	0.52
RMSE	0.61
R^2^	0.57

^1^ Showing the predictors selected by the LASSO model; see full set of eye gaze metrics in Table 1; The R^2^ presents the explained variance by the model, while MAE and RMSE represent a measure of the error of the model.

**Table 4:** Comparison of the model-based clusters of eye movements. The comparison was conducted based on the transition matrices or the Bayes Factor for each cluster.

Metric	Cluster 1	Cluster 2	Analysis
Gaze direction	Row-wise	Row-wise and Column-wise	Transition matrices (see supplementary file)
Probability of transition to answer choices from top or middle row	Low	Low to moderate	Transition matrices probabilities (see supplementary file)
Average time in each test item	-	-	BF_10_ = 0.07 (±<0.00) ^oo^
# Matrix–matrix transitions	-	-	BF_10_ = 0.40 (±<0.00)
# Matrix–answer transitions	-	-	BF_10_ = 0.06 (±<0.00) ^oo^
# Answer–answer transitions	Fewer transitions	More transitions	BF_10_ = 72.79 (±<0.00) **
Latency to the first fixation on an answer choice	-	-	BF_10_ = 0.06 (±<0.00) ^oo^
Ratio of time spent on the matrix vs. answer choices	-	-	BF_10_ = 0.08 (±<0.00) ^oo^
# Visits to a given matrix cell	-	-	BF_10_ = 0.32 (±<0.00) ^o^
# Visits to a given incorrect answer choice	-	-	BF_10_ = 0.83 (±<0.00)
# Fixations on matrix cells	-	-	BF_10_ = 0.08 (±<0.00) ^oo^
Average fixation duration for a matrix cell	Shorter fixations	Longer fixations	BF_10_ > 1000 (±<0.00) ***
# Fixations on answer choices	-	-	BF_10_ = 1.91 (±<0.00)
Average fixation duration for an answer choice	Shorter fixations	Longer fixations	BF_10_ > 1000 (±<0.00) ***
Rate of matrix–answer transitions	-	-	BF_10_ < 0.16 (±<0.00) ^o^

Note: H1: difference between the clusters; H0: no difference between the clusters. ** Strong evidence for H1; *** extreme evidence for H1; ^o^ moderate evidence for H0; ^oo^ strong evidence for H0.

**Table 5:** Correlation matrix of the cognitive measures and eye-tracking metrics in Study 2.

Variables	1.	2.	3.	4.	5.	6.	7.	8.	9.	10.	11.	12.	13.	14.	15.	16.	17.	18.	19.	20.	21.
1.TowerofLondonscore	1.00																				
2.Corsiscore	0.16	1.00																			
3.WSCTPerseverativeerrors	−0.01	−0.04	1.00																		
4.BRIEF−ABRI	−0.18	0.12	0.11	1.00																	
5.BRIEF−AMCI	−0.07	0.15	0.11	0.51 ***	1.00																
6.BRIEF−AGEC	−0.13	0.16	0.13	0.81 ***	0.91 ***	1.00															
7.WMT−2totalscore	0.50 ***	0.31 **	−0.28 *	−0.23	−0.11	−0.18	1.00														
8.Averagetimeineachtestitem	0.19	−0.27 *	−0.00	−0.30 *	−0.09	−0.21	0.31 *	1.00													
9.#Matrix−matrixtransitions	0.16	−0.28 *	0.13	−0.30 *	−0.17	−0.26 *	0.35 **	0.75 ***	1.00												
10.#Matrix−answertransitions	0.03	−0.37 **	0.21	−0.20	−0.12	−0.17	−0.10	0.62 ***	0.58 ***	1.00											
11.#Answer−answertransitions	−0.02	−0.41 ***	0.19	−0.19	−0.13	−0.18	−0.02	0.66 ***	0.74 ***	0.85 ***	1.00										
12.Latencytothefirstfixationonananswerchoice	0.37 **	0.10	−0.20	−0.14	−0.05	−0.10	0.32 **	0.29 *	−0.01	−0.27 *	−0.28 *	1.00									
13.Ratiooftimespentonthematrixvsanswerchoices	0.26 *	0.11	−0.15	−0.26 *	−0.15	−0.22	0.36 **	0.05	0.07	−0.26 *	−0.38 **	0.53 ***	1.00								
14.#Visitstoagivenmatrixcell	0.15	−0.30 *	0.15	−0.30 *	−0.17	−0.26 *	0.31 **	0.77 ***	1.00 ***	0.66 ***	0.79 ***	−0.04	0.03	1.00							
15.#Visitstoagivenincorrectanswerchoice	−0.03	−0.43 ***	0.21	−0.19	−0.12	−0.17	−0.11	0.67 ***	0.67 ***	0.94 ***	0.97 ***	−0.27 *	−0.36 **	0.73 ***	1.00						
16.#Fixationsonmatrixcells	0.17	−0.30 *	0.11	−0.32 **	−0.18	−0.27 *	0.34 **	0.84 ***	0.98 ***	0.63 ***	0.74 ***	0.08	0.09	0.98 ***	0.70 ***	1.00					
17.Averagefixationdurationforamatrixcell	−0.25 *	−0.07	0.07	0.09	0.04	0.07	−0.21	−0.19	−0.16	−0.11	0.00	−0.12	−0.18	−0.16	−0.04	−0.22	1.00				
18.#Fixationsonanswerchoices	0.01	−0.40 ***	0.17	−0.20	−0.11	−0.17	−0.02	0.72 ***	0.74 ***	0.92 ***	0.98 ***	−0.24 *	−0.33 **	0.80 ***	0.99 ***	0.77 ***	−0.08	1.00			
19.Averagefixationdurationforananswerchoice	−0.20	−0.11	0.19	0.16	0.16	0.19	−0.18	−0.10	−0.03	0.06	0.17	−0.26 *	−0.37 **	−0.02	0.13	−0.11	0.86 ***	0.08	1.00		
20.Percentoftrialsclassifiedascluster2scanpath	−0.03	−0.16	0.18	0.10	0.13	0.14	−0.02	−0.07	0.17	0.07	0.24 *	−0.09	−0.12	0.16	0.17	0.08	0.41 ***	0.17	0.41 ***	1.00	
21.Rateofmatrix−answertransitions	−0.21	−0.23	0.28 *	0.11	0.01	0.06	−0.50 ***	−0.26 *	−0.11	0.51 ***	0.29 *	−0.69 ***	−0.46 ***	−0.04	0.39 ***	−0.15	0.04	0.31 **	0.16	0.08	1.00

*Note:* **Bold** values indicate significant correlations. * *p* < 0.05; ** *p* < 0.01; *** *p* < 0.001.

**Table 6:** Standardized coefficients and performance estimates of the LASSO regression model predicting cognitive test performance and self-reported EF.

Measures	Corsi	TOL Score	TOL Time	WSCT Perseverative Errors	BRIEF-A BRI	BRIEF-A MCI	BRIEF-A GEC
Predictor’s standardized coefficients ^1^							
Average time in each test item	-	-	−0.65	-	−0.09	-	-
Matrix–answer transitions	-	-	−2.00	-	-	-	-
Answer-answer transitions	-	-	−3.66	-	-	-	-
Latency to first fixation in answer choices	-	0.14	−0.22	-	-	-	-
Ratio of time spent on matrix vs. answers	-	0.02	−0.16	-	−0.10	-	-
Visits to a given matrix cell	-	-	−0.96	-	-	-	-
Visits in wrong answer choices	−0.27	-	3.38	-	-	-	-
Total number of fixations on matrix cells	-	-	1.83	-	-	-	-
Average fixation duration for a matrix cell	-	−0.13	0.02	-	−0.14	-	-
Total number of fixations on answer choices	-	-	2.13	-	-	-	-
Average fixation duration for an answer choice	-	-	0.30	-	0.03	-	-
Percent of trials classified as cluster 2 scanpath	−0.03	-	−0.40	-	-	-	-
Rate of matrix–answer transitions	-	-	−0.18	-	-	-	-
Performance estimates							
Correlation coefficient	0.48	0.59	0.14	-	0.09	-	-
MAE	0.58	0.69	0.94	-	0.73	-	-
RMSE	0.68	0.92	1.11	-	0.95	-	-
R^2^	0.18	0.16	−2.13	-	−0.03	-	-

^1^ Showing the predictors selected by the LASSO model; see full set of eye gaze metrics in Table 1; The R^2^ presents the explained variance by the model, while MAE and RMSE represent measures of the error of the model; BRI = behavior regulation Index; MCI = Metacognition Index; GEC = Global Executive Composite.

## Data Availability

The data presented in this study are openly available in Open Science Framework (OSF) at https://osf.io/38fuy/, doi: 10.17605/OSF.IO/38FUY.

## Supplementary Materials

The following supporting information can be downloaded at: https://www.mdpi.com/article/10.3390/jintelligence11040075/s1, S1: Changes after preregistering the analysis; Figure S1: Scree plot of the clusterization model of Study 1; Figure S2: Average transition matrix plot of the clusters of Study 1; Figure S3: Representatives of the eye movements categorized in each cluster of Study 1; Figure S4: Raincloud plots for the eye-tracking metric variables used in the Study 1; Table S1. Descriptives of each cluster; Table S2. Correlation matrix of the cognitive measures and eyetracking metrics in Study 1; Table S3. Coefficients and measures of the LASSO regression model predicting the WMT-2; Figure S5: Scree plot of the clusterization model of Study 2; Figure S6: Average transition matrix plot of the clusters of Study 2; Figure S7: Representatives of the eye movements categorized in each cluster of Study 2; Figure S8: Raincloud plots for the eye-tracking metric variables used in the Study 2; Table S4. Descriptives of each cluster; Table S5. Coefficients and measures of the LASSO regression model predicting the WMT-2.
