# Supplementary material for: Eye Gaze Patterns during Reasoning Provide Insights Regarding Individual Differences in Underlying Cognitive Abilities
Source: J Intell. 2023 Apr 20;11(4):75. doi: 10.3390/jintelligence11040075 (PMC10145645; doi:10.3390/jintelligence11040075)
Supplement: Supplementary file 1 [file jintelligence-11-00075-s001.zip › jintelligence-2158535-supplementary.pdf]

Eye gaze patterns during reasoning provide insights regarding individual differences in underlying cognitive abilities – supplementary information

Summary:

1. **Changes in the preregistered plan.**

S1. Changes after preregistering the analysis.

2. **Study 1**

Figure S1. Scree plot of the clusterization model.

Figure S2. Average transition matrix plot of the clusters.

Figure S3. Representatives of the eye movements categorized in each cluster.

Figure S4. Raincloud plots for the eyetracking metric variables used in the Study 1.

Table S1. Descriptives of each cluster.

Table S2. Correlation matrix of the cognitive measures and eyetracking metrics in Study 1.

Table S3. Coefficients and measures of the LASSO regression model predicting the WMT-2.

3. **Study 2**

Figure S5. Scree plot of the clusterization model.

Figure S6. Average transition matrix plot of the clusters.

Figure S7. Representatives of the eye movements categorized in each cluster.

Figure S8. Raincloud plots for the eyetracking metric variables used in the Study 2.

Table S4. Descriptives of each cluster.

Table S5. Coefficients and measures of the LASSO regression model predicting the WMT-2.

1. Change in the preregistered plan

S1. Changes after preregistering the analysis.

In the last paragraph of Question 5 in the AsPredicted, we mentioned that we would do exploratory analysis to predict the performance in the different cognitive tests. However, we decided to use a Least Absolute Shrinkage and Selection Operator (LASSO) regularization (L1) instead of the Elastic-Net regularization (L1+L2). Our change occurred because we were interested in selecting different eye movement predictors and the L2 regularization would not help with it. Therefore, the penalization without exclusion of any of the independent variables by the L2 regularization would not be useful to us. Consequently, we decided to only use the L1 regularization.

Furthermore, in Question 2 of the AsPredicted document, we listed several eye gaze metrics that were used as predictors of the cognitive tests in Study 1 and 2. However, after discussion with the reviewers, we agreed that the "**Rate of Toggling**" metric (first mentioned in Vigneau et al., Intelligence, 2006) was an important eye gaze variable, due to its relevance in the literature and because it is the best predictor in several other manuscripts (e.g., Hayes et al., Journal of Vision, 2011; Laurence et al., Frontiers in Psychology, 2018). Therefore, we chose to add this variable to our models, calling it as "**Rate of Matrix-Answer transitions**" (so that the variable name is more accurate), even though it was not mentioned in the pre-registration.

2. Study 1

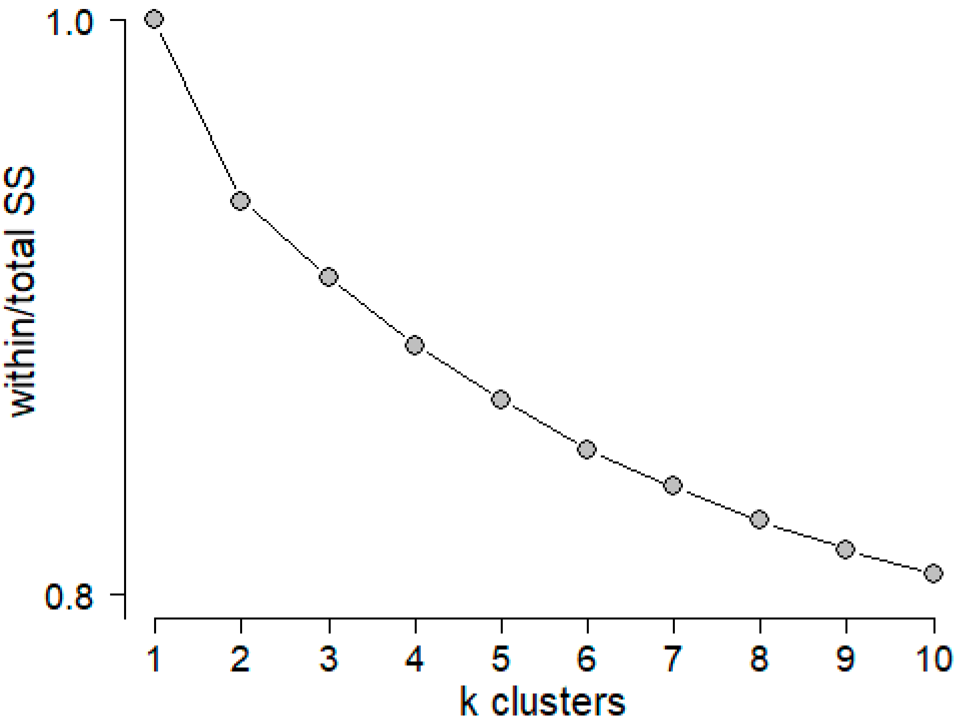

Figure S1. The scree plot of the clusterization model. In this plot, it is possible to see that there is no evident "elbow" on the plot. Therefore, since the literature is based on 2 strategies (e.g., constructive matching and response elimination), we opted to use k = 2.

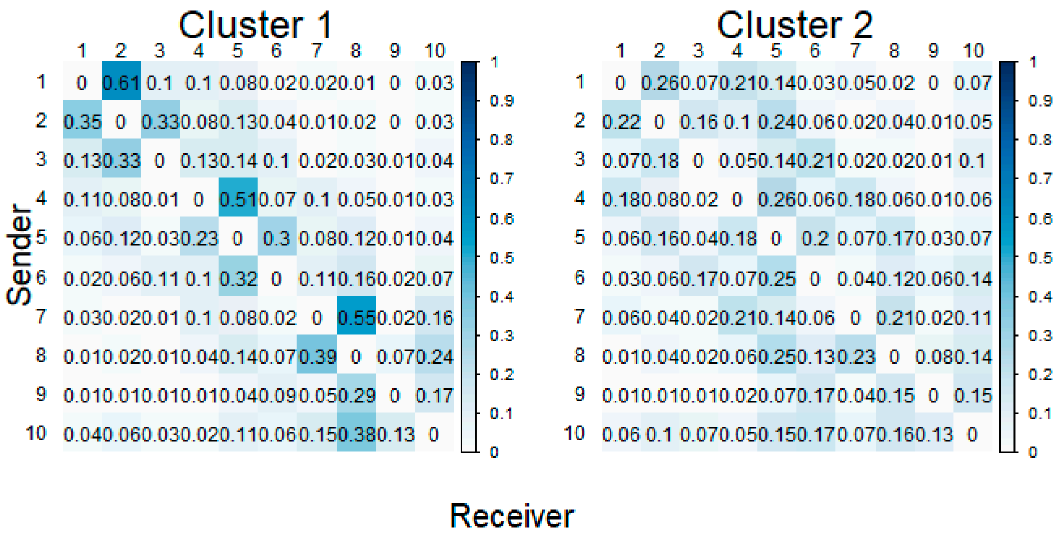

Figure S2. Average transition matrix plot of the clusters. Each row is the sender, and each column is the receiver. Higher values (and blueish colors) imply a higher probability that the fixation that occurred in that row will go to the cell indicate by the column. Cluster 1 tends fixations in each line of the matrix task, while cluster 2 has a tendency of line and columns. Cluster 2 also has a higher chance of going to the answers (AOI 10) while under cells 1 to 6.

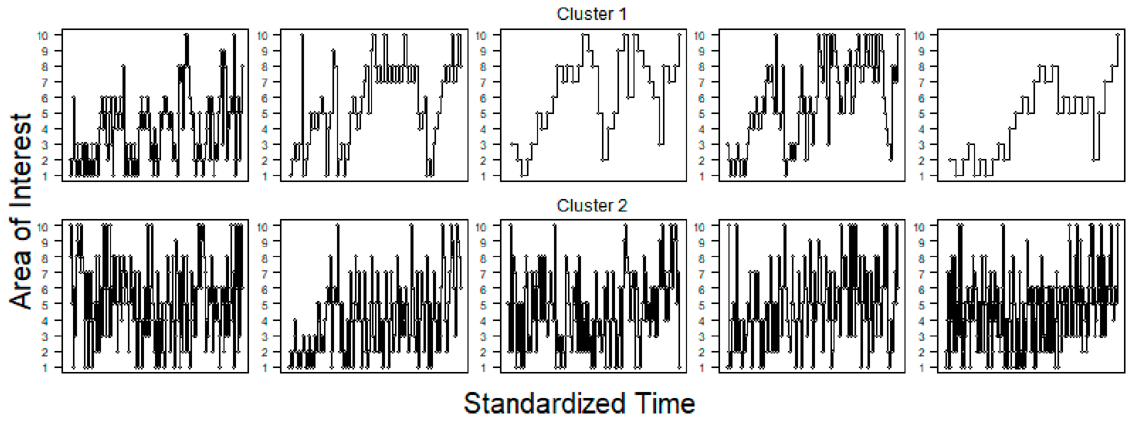

Figure S3. Representatives of the eye movements categorized in each cluster. Each plot is the sequence of fixations in the AOIs while in one trial of the matrix reasoning task.

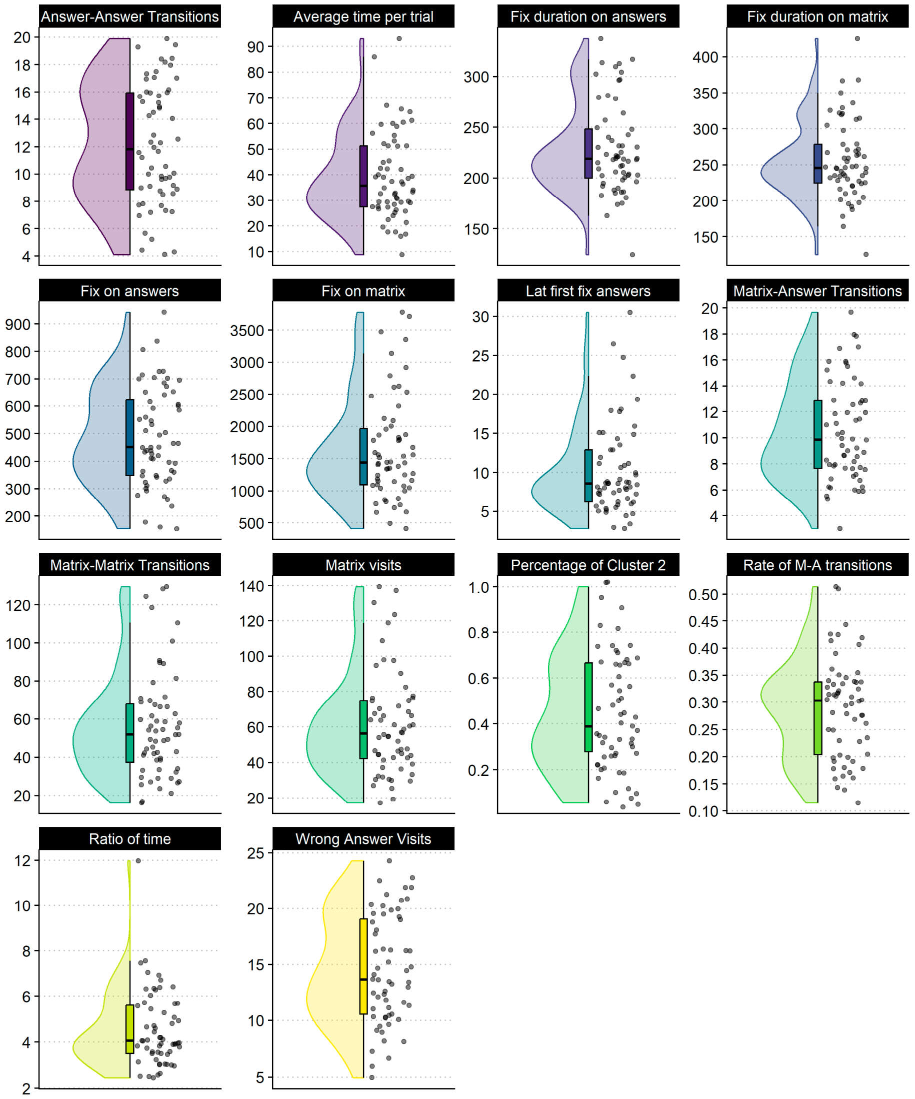

Figure S4. Raincloud plots for the eyetracking metric variables used in the Study 1. The dots indicate the given test score of each participant, and the box and violin plots present the data distribution.

Table S1. Descriptive statistics of each cluster regarding the eyetracking metrics in Study 2. **Bold** lines indicate significant difference in the Bayesian tests.

Metric	Cluster 1 Mean (SD)	Cluster 2 Mean (SD)
Average time in each test item (in seconds)	39.24 (29.08)	39.59 (31.75)
# Matrix-matrix transitions	59.20 (44.18)	54.86 (48.71)
# Matrix-answer transitions	10.46 (7.78)	10.72 (8.23)
# **Answer-answer transitions**	**13.43 (10.76)**	**11.45 (9.41)**
Latency to the first fixation on an answer choice (in seconds)	11.17 (12.45)	9.42 (11.43)
Ratio of time spent on the matrix vs answer choices	0.77 (0.09)	0.77 (0.11)
# Visits to a given matrix cell	64.11 (46.71)	59.95 (51.18)
# Visits to a given incorrect answer choice	15.34 (11.84)	13.97 (10.92)
# Fixations on matrix cells	91.72 (70.18)	92.37 (81.74)
# Fixations on answer choices	28.69 (21.31)	26.31 (20.18)
**Average fixation duration for a matrix cell (in ms)**	**273.85 (64.42)**	**243.79 (59.71)**
**Average fixation duration for an answer choice (in ms)**	**240.96 (59.02)**	**219.80 (61.11)**
**Rate of Matrix-answer transitions**	**0.326 (0.186)**	**0.293 (0.151)**

Table S2. Correlation matrix of the cognitive measures and eyetracking metrics in Study 1.

Variables	1.	2.	3.	4.	5.	6.	7.	8.	9.	10.	11.	12.	13.	14.	15.	16.
1. D.70 total score	1.00															
2. WMT-2 total score	0.54***	1.00														
3. Average time in each test item	-0.09	0.43***	1.00													
4. # Matrix-Matrix transitions	0.03	0.49***	0.86***	1.00												
5. # Matrix-answer transitions	-0.32*	0.10	0.65***	0.66***	1.00											
6. # Answer-answer transitions	-0.12	0.24	0.72***	0.74***	0.77***	1.00										
7. Latency to the first fixation on an answer choice	0.06	0.32*	0.56***	0.44***	-0.03	0.12	1.00									
8. Ratio of time spent on the matrix vs answer choices	0.14	0.41**	0.50***	0.51***	-0.00	0.01	0.60***	1.00								
9. # Visits to a given matrix cell	0.01	0.47***	0.87***	1.00***	0.70***	0.76***	0.42***	0.49***	1.00							
10. # Visits to a given incorrect answer choice	-0.25	0.13	0.72***	0.71***	0.89***	0.96***	0.05	-0.03	0.74***	1.00						
11. # Fixations on matrix cells	0.05	0.48***	0.87***	0.97***	0.63***	0.72***	0.43***	0.52***	0.97***	0.69***	1.00					
12. Average fixation duration for a matrix cell	-0.03	0.06	0.20	0.09	0.12	0.15	0.21	0.14	0.10	0.14	-0.05	1.00				
13. # Fixations on answer choices	-0.14	0.21	0.69***	0.73***	0.77***	0.93***	0.07	0.02	0.75***	0.92***	0.78***	-0.07	1.00			
14. Average fixation duration for an answer choice	-0.02	0.07	0.20	0.07	0.20	0.14	0.13	-0.03	0.08	0.17	-0.07	0.91***	-0.07	1.00		
15. Percent of trials classified as cluster 2 scanpath	-0.03	-0.03	0.00	-0.20	-0.10	-0.27*	-0.10	0.04	-0.20	-0.20	-0.07	-0.41***	-0.14	-0.32*	1.00	
16. Rate of matrix-answer transitions	-0.35**	-0.53***	-0.56***	-0.39**	0.19	-0.16	-0.68***	-0.60***	-0.36**	-0.03	-0.42***	-0.11	-0.10	-0.02	-0.04	1.00

Note: **Bold** values indicates significant correlations. *p<0.05; **p<0.01; ***p<0.001

Table S3. Coefficients and measures of the LASSO regression model predicting the WMT-2 total score of the sample in Study 1.

Measures	Standardized Coefficients
Predictors [1]	
Average time in each test item	-0.20
Matrix-answer transitions	0.96
Answer-answer transitions	1.40
Latency to first fixation in answer choices	-0.22
Visits to a given matrix cell	0.40
Visits in wrong answer choices	-2.21
Total number of fixations on answer choices	0.06
Average fixation duration for an answer choice	0.03
Percent of trials classified as cluster 2 scanpath	0.03
Rate of Matrix-Answer transitions	-0.63
Performance estimates	
Correlation coefficient	0.68
MAE	0.70
RMSE	0.84
R^2	0.44

[1] Showing the predictors selected by the LASSO model; see full set of eye gaze metrics in Table 1 of the manuscript; The R^2 presents the explained variance by the model, while MAE and RMSE represent a measure of the error of the model.

3. Study 2

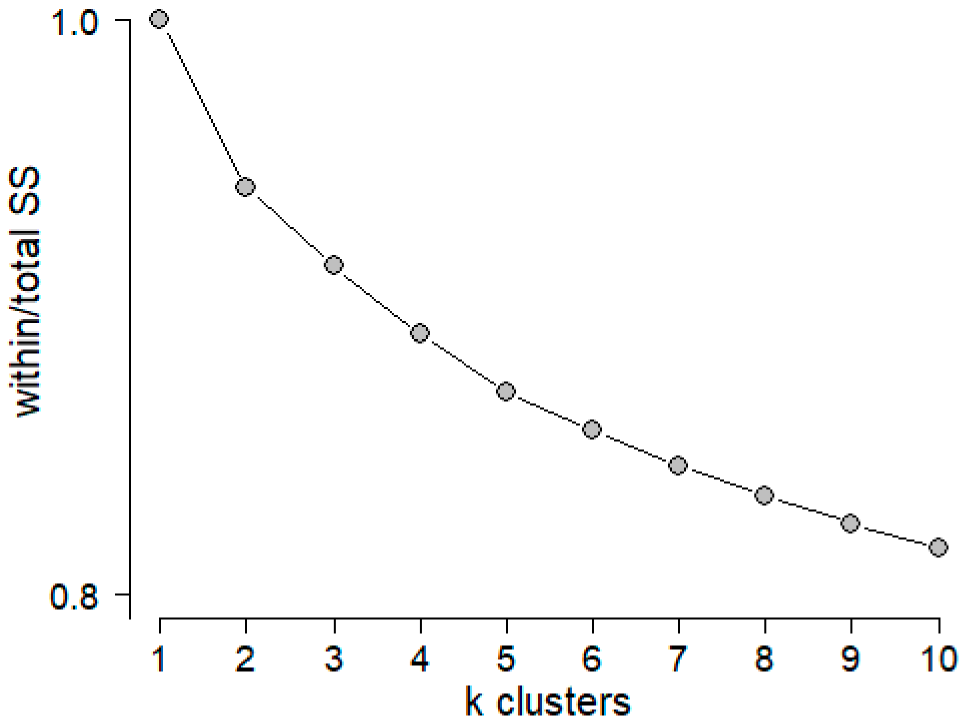

Figure S5. The scree plot of the clusterization model. Similar to the scree plot of study 1 (Figure S1), it is not possible to see a clear “elbow”. Therefore, we followed the same concept of Study 1 and used k = 2 based on the literature.

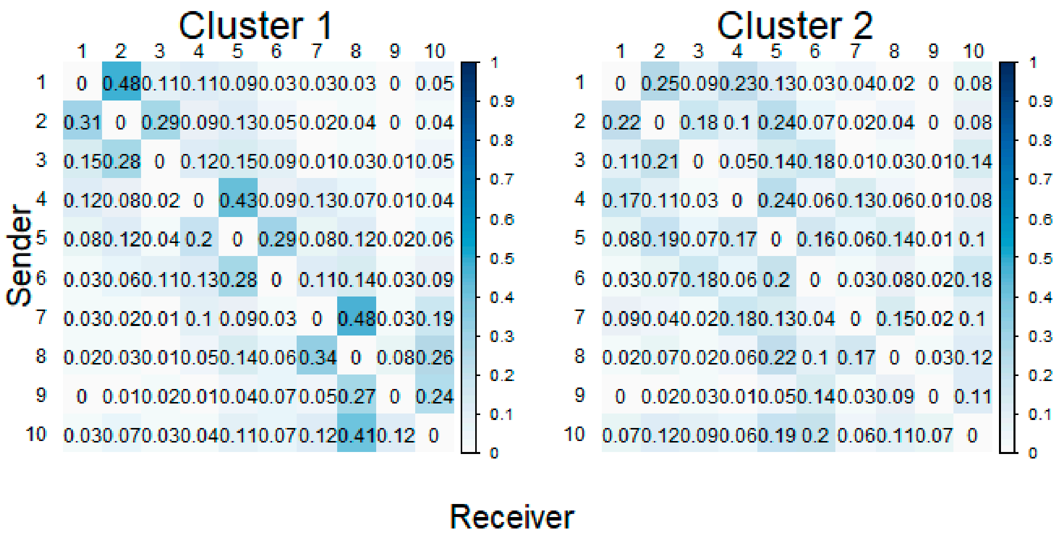

Figure S6. Average transition matrix plot of the clusters. Each row is the sender, and each column is the receiver. Higher values (and blueish colors) imply a higher probability that the fixation that occurred in that row will go to the cell indicate by the column. Similar to the data found in Study 1, cluster 1 has a tendency of fixations in each line of the matrix task, while cluster 2 has a tendency of line and columns. Cluster 2 also has a higher chance of going to the answers (AOI 10) while under cells 1 to 6.

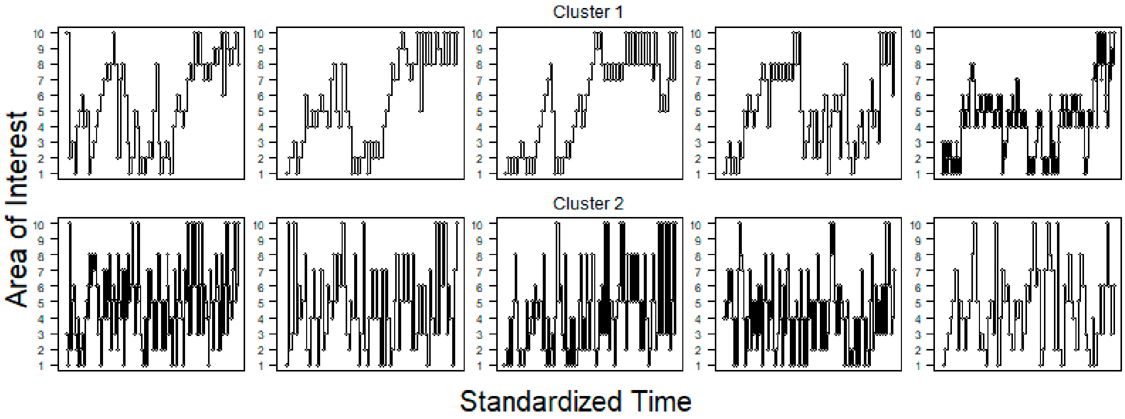

Figure S7. Representatives of the eye movements categorized in each cluster. Each plot is the sequence of fixations in the AOIs while in one trial of the matrix reasoning task.

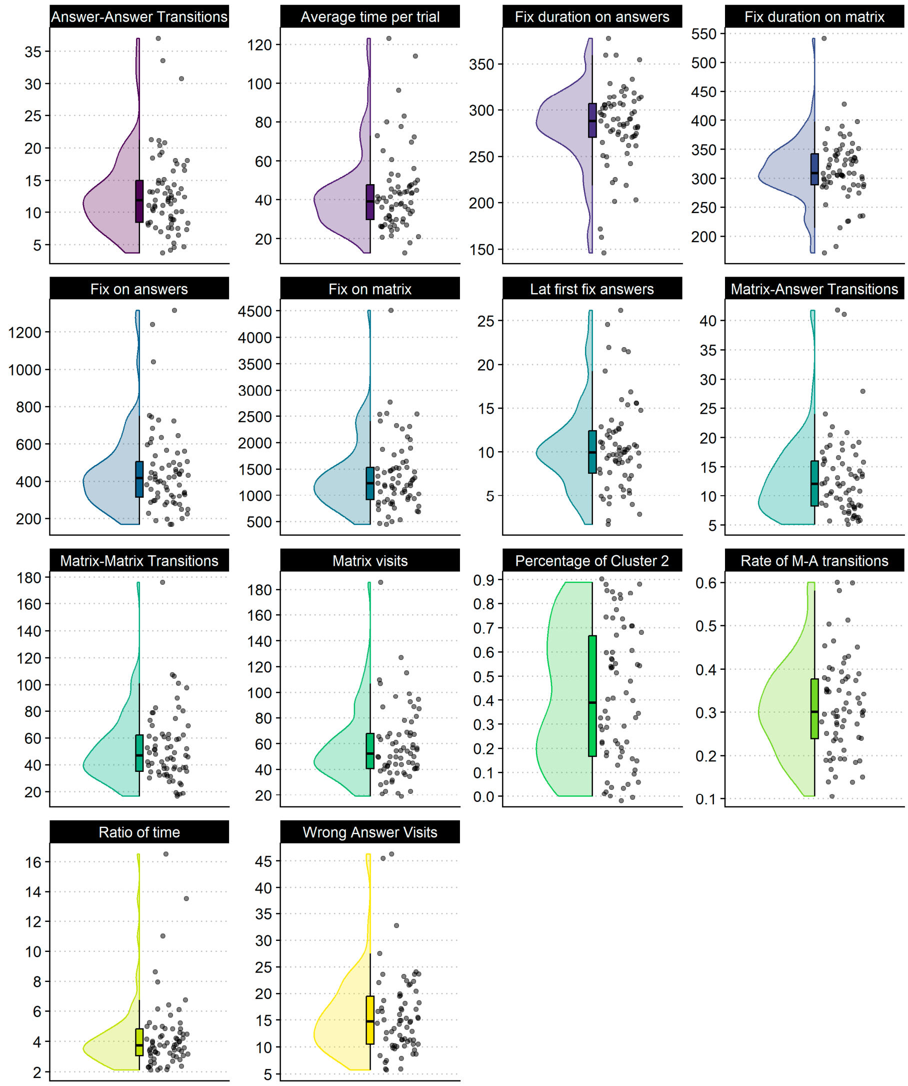

Figure S8. Raincloud plots for the eyetracking metric variables used in the Study 2. The dots indicate the given test score of each participant, and the box and violin plots present the data distribution.

Table S4. Descriptive statistics of each cluster regarding the eyetracking metrics in Study 2. **Bold** lines indicate significant difference in the Bayesian tests.

Metric	Cluster 1 Mean (SD)	Cluster 2 Mean (SD)
Average time in each test item (in seconds)	44.29 (42.39)	43.24 (32.76)
# Matrix-matrix transitions	50.25 (55.11)	55.92 (44.59)
# Matrix-answer transitions	13.17 (10.98)	13.18 (11.07)
# **Answer-answer transitions**	**11.86 (10.92)**	**14.33 (11.82)**
Latency to the first fixation on an answer choice (in seconds)	10.49 (11.92)	10.55 (10.89)
Ratio of time spent on the matrix vs answer choices	0.76 (0.12)	0.75 (0.09)
# Visits to a given matrix cell	56.61 (58.12)	62.25 (48.39)
# Visits to a given incorrect answer choice	15.16 (13.27)	16.94 (13.88)
# Fixations on matrix cells	75.43 (81.26)	78.28 (61.76)
# Fixations on answer choices	23.81 (20.09)	26.92 (21.12)
**Average fixation duration for a matrix cell (in ms)**	**300.35 (66.49)**	**335.40 (73.59)**
**Average fixation duration for an answer choice (in ms)**	**271.79 (66.54)**	**298.52 (66.75)**
Rate of Matrix-answer transitions	0.345 (0.195)	0.331 (0.166)

Table S5. Coefficients and measures of the LASSO regression model predicting the WMT-2 total score of the sample in Study 2.

Measures	Standardized Coefficients
Predictors [1]	
Average time in each test item	0.24
Matrix-answer transitions	1.23
Answer-answer transitions	2.18
Latency to first fixation in answer choices	-0.03
Ratio of time	0.13
Visits to a given matrix cell	0.08
Visits in wrong answer choices	-3.72
Mean fixation duration in matrix	-0.31
Total number of fixations on answer choices	0.22
Mean fixation duration on answer choices	0.17
Percent of trials classified as cluster 2 scanpath	-0.01
Rate of Matrix-answer transitions	-0.25
Performance estimates	
Correlation coefficient	0.78
MAE	0.53
RMSE	0.63
R^2	0.59

[1] Showing the predictors selected by the LASSO model; see full set of eye gaze metrics in Table 1 of the manuscript; The R^2 presents the explained variance by the model, while MAE and RMSE represent a measure of the error of the model.
